# Genomic regions and biological mechanisms underlying climatic resilience traits derived from automatically-recorded vaginal temperature in lactating sows under heat stress conditions

**DOI:** 10.3389/fgene.2024.1498380

**Published:** 2024-11-07

**Authors:** Hui Wen, Jay S. Johnson, Henrique A. Mulim, Andre C. Araujo, Felipe E. De Carvalho, Artur O. Rocha, Yijian Huang, Francesco Tiezzi, Christian Maltecca, Allan P. Schinckel, Luiz F. Brito

**Affiliations:** ^1^ Department of Animal Sciences, Purdue University, West Lafayette, IN, United States; ^2^ Division of Animal Sciences, College of Agriculture, Food and Natural Resources, University of Missouri, Columbia, MO, United States; ^3^ Smithfield Premium Genetics, Raleigh, NC, United States; ^4^ Department of Agriculture, Food, Environment and Forestry, University of Florence, Firenze, Italy; ^5^ Department of Animal Science, North Carolina State University, Raleigh, NC, United States

**Keywords:** climate resilience, heat stress, genome-wide association studies, livestock breeding, genomic regions

## Abstract

Climate change poses a growing threat to the livestock industry, impacting animal productivity, animal welfare, and farm management practices. Thus, enhancing livestock climatic resilience (CR) is becoming a key priority in various breeding programs. CR can be defined as the ability of an animal to be minimally affected or rapidly return to euthermia under thermally stressful conditions. The primary study objectives were to perform genome-wide association studies for 12 CR indicators derived from variability in longitudinal vaginal temperature in lactating sows under heat stress conditions. A total of 31 single nucleotide polymorphisms (SNPs) located on nine chromosomes were considered as significantly associated with nine CR indicators based on different thresholds. Among them, only two SNPs were simultaneously identified for different CR indicators, SSC6:16,449,770 bp and SSC7:39,254,889 bp. These results highlighted the polygenic nature of CR indicators with small effects distributed across different chromosomes. Furthermore, we identified 434 positional genes associated with CR. Key candidate genes include *SLC3A2*, *STX5*, *POLR2G*, and *GANAB*, which were previously related to heat stress responses, protein folding, and cholesterol metabolism. Furthermore, the enriched KEGG pathways and Gene Ontology (GO) terms associated with these candidate genes are linked to stress responses, immune and inflammatory responses, neural system, and DNA damage and repair. The most enriched quantitative trait loci are related to “Meat and Carcass”, followed by “Production”, “Reproduction”, “Health”, and “Exterior (conformation and appearance)” traits. Multiple genomic regions were identified associated with different CR indicators, which reveals that CR is a highly polygenic trait with small effect sizes distributed across the genome. Many heat tolerance or HS related genes in our study, such as *HSP90AB1*, *DMGDH*, and *HOMER1*, have been identified. The complexity of CR encompasses a range of adaptive responses, from behavioral to cellular. These results highlight the possibility of selecting more heat-tolerant individuals based on the identified SNP for CR indicators.

## 1 Introduction

Climate change poses an increasing risk to productivity, welfare, and requires farm management practices change without compromising effectiveness in the livestock industry (Rhoads et al., 2009; Yang et al., 2021). Moreover, economically important traits are significantly influenced by genotype-by-environment interactions, and the animals’ performance can deteriorate with rising global temperatures (Haile-Mariam et al., 2008; Braz et al., 2021). Over the past few decades, intensive breeding for greater productivity, such as increased milk yield, growth rate, little size, and body weight in livestock, led to higher metabolic heat production (Cabezón et al., 2017), potentially reducing the ability of livestock (i.e., pigs) to thrive in harsh environments. Consequently, this has caused farm management to become more challenging with declining profitability as animals face heightened risks from the severe effects of global warming. Thus, enhancing CR has become a primary objective in livestock breeding.

Climate resilience refers to the animal’s ability to maintain or quickly return to euthermia under thermally stressful conditions (Colditz and Hine, 2016; Wen et al., 2024). Many studies have investigated CR and proposed novel indicators, such as genetic variance of the slope of reaction norm models (Shi et al., 2021; Waters et al., 2022; Freitas et al., 2024). However, most direct resilience phenotypes are difficult or expensive to measure, leading to less frequent measurements and more issues, such as low phenotypic variability and low to moderate heritability estimates (Guy et al., 2012; Gorssen et al., 2021). In previous studies, various phenotypes related to heat stress (HS) in lactating sows—including vaginal temperature, respiration rate, skin surface temperature, hair density, and body condition score—were measured under HS conditions and considered as useful indicators of HS (Scheffer et al., 2018; Gorssen et al., 2021; Johnson et al., 2023). These phenotypes exhibited low to moderate heritability estimates. However, identifying CR of animals based on these measures alone is challenging. Individuals who exhibit greater consistency in their phenotypes over time are likely to have higher resilience (Scheffer et al., 2018; Berghof et al., 2019). This is because they are expected to deviate less from their optimal production or physiological levels when faced with disruptions, leading to increased survival and reduced disease incidence (Scheffer et al., 2018; Berghof et al., 2019).

Increased availability of longitudinal data from various methods, such as automatic thermometers, feeding stations, and computer vision systems, makes it possible to derive more effective resilience indicators (Chen et al., 2023; Pedrosa et al., 2023). For instance, methods for deriving several new resilience indicators in dairy cattle based on deviations from observed and expected performance, including variance, lag-1 autocorrelation, and skewness of deviations, have been proposed (Poppe et al., 2020). These methods have been applied in resilience studies across various species, including cattle (Poppe et al., 2020; Chen et al., 2023), pigs (Mancin et al., 2024), and dairy goats (Sánchez-Molano et al., 2019).

Fifteen novel CR indicators, such as variance, lag-1 autocorrelation, and skewness of deviations, as well as HS duration, using longitudinal automatically-recorded vaginal temperature were developed in our previous study (Wen et al., 2024). Most of these indicators were moderately heritable and had low to high genetic correlations with each other. Current understanding of the biological mechanisms and genetic factors influencing CR in lactating sows is rather limited. In this context, genome-wide association studies (GWAS) enable the detection of single nucleotide polymorphisms (SNP) associated with traits of interest (Visscher et al., 2012). Many GWAS studies focusing on resilience have been conducted in different species, such as chicken (Doekes et al., 2023), pigs (Putz et al., 2019), sheep (Tsartsianidou et al., 2021), and cattle (Alonso-Hearn et al., 2022; Chen et al., 2024). However, CR is expected to be a polygenic trait influenced by numerous biological mechanisms, which could lead to the identification of many putative quantitative trait loci (QTL), some of them with small effect and located on different chromosomes. GWAS can contribute to a better understanding of the genetic basis underlying phenotypic variability in CR. By undertaking GWAS on different CR metrics, we can delve deeper into the genetic basis of this complex trait, potentially uncovering valuable insights that will not only advance our scientific knowledge but also inform breeding strategies aimed at enhancing CR in sows. Thus, the primary study objectives were to 1) detect SNPs and genomic regions significantly associated with twelve CR indicators derived from automatically-recorded vaginal temperature measured in lactating sows under HS conditions; and 2) identify the underlying biological functions and metabolic pathways these regions are involved in based on functional genomic analyses.

## 2 Materials and methods

### 2.1 Datasets

All live animal data collection procedures were approved by the Purdue University Animal Care and Use Committee (Protocol #1912001990). All data collection procedures, physiological data, genotype information, and quality control processes have been previously described in our previous studies (Johnson et al., 2023; Wen et al., 2023; Wen et al., 2024). In brief, 1,639 lactating sows (parities 2–7; Landrace × Large White) were genotyped using the PorcineSNP50K Bead Chip (Illumina, San Diego, CA, United States). The vaginal temperature (T_V_) of 1,381 sows within the studied population was automatically measured every 10 min from June 5th to July 30th, 2021, using a vaginally implanted thermochron data recorder (Johnson et al., 2023). Ambient temperature and humidity of each barn was automatically collected every 5 minutes (Johnson et al., 2023). The phenotypic and genomic quality control procedures performed can be accessed on our research (Johnson et al., 2023; Wen et al., 2023). Twelve CR indicators were derived based on variability in automatically measured vaginal temperature (Wen et al., 2024). Log-transformed variance [LnVar(Ave) and LnVar(Med)], Lag-1 autocorrelation (Autocor (Ave) and Autocor(Med)], and skewness [Skew(Ave) and Skew(Med)] of the deviations between observed and the average (Ave) or median (Med) values from moving windows consisting of six consecutive observations with a 10-minute interval were calculated for each animal (Poppe et al., 2020). The HS thresholds for individuals under distinct ventilation conditions (mechanical ventilation at 39.76°C and natural ventilation at 39.78°C) were reported (Johnson et al., 2023). Additional traits were derived including the daily maximum vaginal temperature (Max_Tv_) per individual and the HS duration (HSD), which quantifies the duration of the time interval in which an individual’s vaginal temperature consistently exceeded the HS threshold each day. Two CR indicators were normalized median ( Nor_medvar ) or average T_V_ ( Nor_avevar ) multiplied by the normalized T_V_ variance on a population level as follows,

${\text{Nor}\_\text{medvar}}_{i}=\frac{{\text{Med}}_{i}-{\text{Med}}_{\min}}{{\text{Med}}_{\max}-{\text{Med}}_{\min}}\times \frac{{\text{var}\left(\text{Tv}\right)}_{i}-{\text{var}\left(\text{Tv}\right)}_{\min}}{{\text{var}\left(\text{Tv}\right)}_{\max}-{\text{var}\left(\text{Tv}\right)}_{\min}}$ and ${\text{Nor}\_\text{avevar}}_{i}=\frac{{\text{Ave}}_{i}-{\text{Ave}}_{\min}}{{\text{Ave}}_{\max}-{\text{Ave}}_{\min}}\times \frac{{\text{var}\left(\text{Tv}\right)}_{i}-{\text{var}\left(\text{Tv}\right)}_{\min}}{{\text{var}\left(\text{Tv}\right)}_{\max}-{\text{var}\left(\text{Tv}\right)}_{\min}}$ , where ${\text{Med}}_{i}$ , ${\text{Ave}}_{i}$ , and ${\text{Var}\left(\text{Tv}\right)}_{i}$ represent the median, average, and variance of T_V_ for individual i , ${\text{Ave}}_{\min}$ and ${\text{Ave}}_{\max}$ are the minimum and maximum median T_V_, ${\text{Med}}_{\min}$ and ${\text{Med}}_{\max}$ are the minimum and maximum median T_V_, and ${\text{Var}\left(\text{Tv}\right)}_{\min}$ and ${\text{Var}\left(\text{Tv}\right)}_{\max}$ are the minimum and maximum T_V_ variance, respectively. Furthermore, two additional traits were derived based on the total deviations between T_V_ and HS threshold values, which were calculated by summing up the T_V_ values above ( ${\text{HSU}}_{A}$ ) or below ( ${\text{HSU}}_{B}$ ) the HS threshold throughout the entire data collection period as follows,

${\text{HSU}}_{A}=\sum_{t=1}^{n}\left({\text{Tv}}_{t}-\text{HS threshold}\right)$ and ${\text{HSU}}_{B}=\sum_{t=1}^{n}\left({\text{Tv}}_{t}-\text{HS threshold}\right)$ , where ${\text{Tv}}_{t}$ is the T_V_ at time point t . All these CR indicators were described in detail (Wen et al., 2024), and the heritability estimates ranged from 0.084 ± 0.037 [Skew (Med)] to 0.291 ± 0.047 (HSU_B_).

### 2.2 Genome-wide association studies and functional genomic analyses

Genome-wide association studies between the CR indicators and the SNPs were conducted using the linear mixed animal model in the GCTA software (Yang et al., 2011), with the option of leaving one chromosome out (MLMA-LOCO). The effects included in the GWAS models are the same as those reported previously (Wen et al., 2024). After performing the GWAS, the genomic inflation factor (λ) was calculated to evaluate potential bias in the results, e.g., from unaccounted population stratification. The λ value was calculated as the ratio of the median of the observed distribution of the statistic to the expected median, for which a 95% confidence interval of value was further derived (Devlin and Roeder, 1999). The Bonferroni correction was used for multiple testing corrections (Armstrong, 2014). The genome-wide significance and suggestive significance threshold were set as $1.17\times 10^{-6}$ (*P* = 0.05/N) and $2.34\times 10^{-5}$ (*P* = 1/N), respectively, where N represents the total SNP number left after removing SNPs based on linkage disequilibrium (LD) (indep-pairwise 50 5 0.1, N = 42,729). To avoid type I errors and false negative results, another less-stringent significance threshold of 0.05 divided by the number of independent chromosomal segments ( $M_{e}$ ) at chromosome-wise levels was considered (Li et al., 2015), following the model: Me=2NeLlog NeL , where $M_{e}$ is an function of effective population size ( $N_{e}$ ) and chromosome length ( L , in centimorgans–cM). $N_{e}$ was considered to be equal to 60 (Hall, 2016) and 1 cM equivalent to 1 Mb (Wang et al., 2016). Quantile-quantile plots (Q-Q plots) were created using the CMplot R package (Yin et al., 2021).

The GALLO R package (Fonseca et al., 2020) was used to detect genes located within 500 Kb up and downstream of significant SNP and QTL regions previously cited in the pig QTLdb (Hu et al., 2019) based on the latest genome reference Sscrofa 11.1 assembly (http://useast.ensembl.org/Sus_scrofa/Info/Index). Gene Ontology (GO) (Ashburner et al., 2000) and Kyoto Encyclopedia of Genes and Genomes (KEGG) (Kanehisa and Goto, 2000) enrichment analyses for candidate genes were carried out using the DAVID platform (Huang et al., 2009).

## 3 Results and discussion

### 3.1 GWAS results summary

**FIGURE 1:**
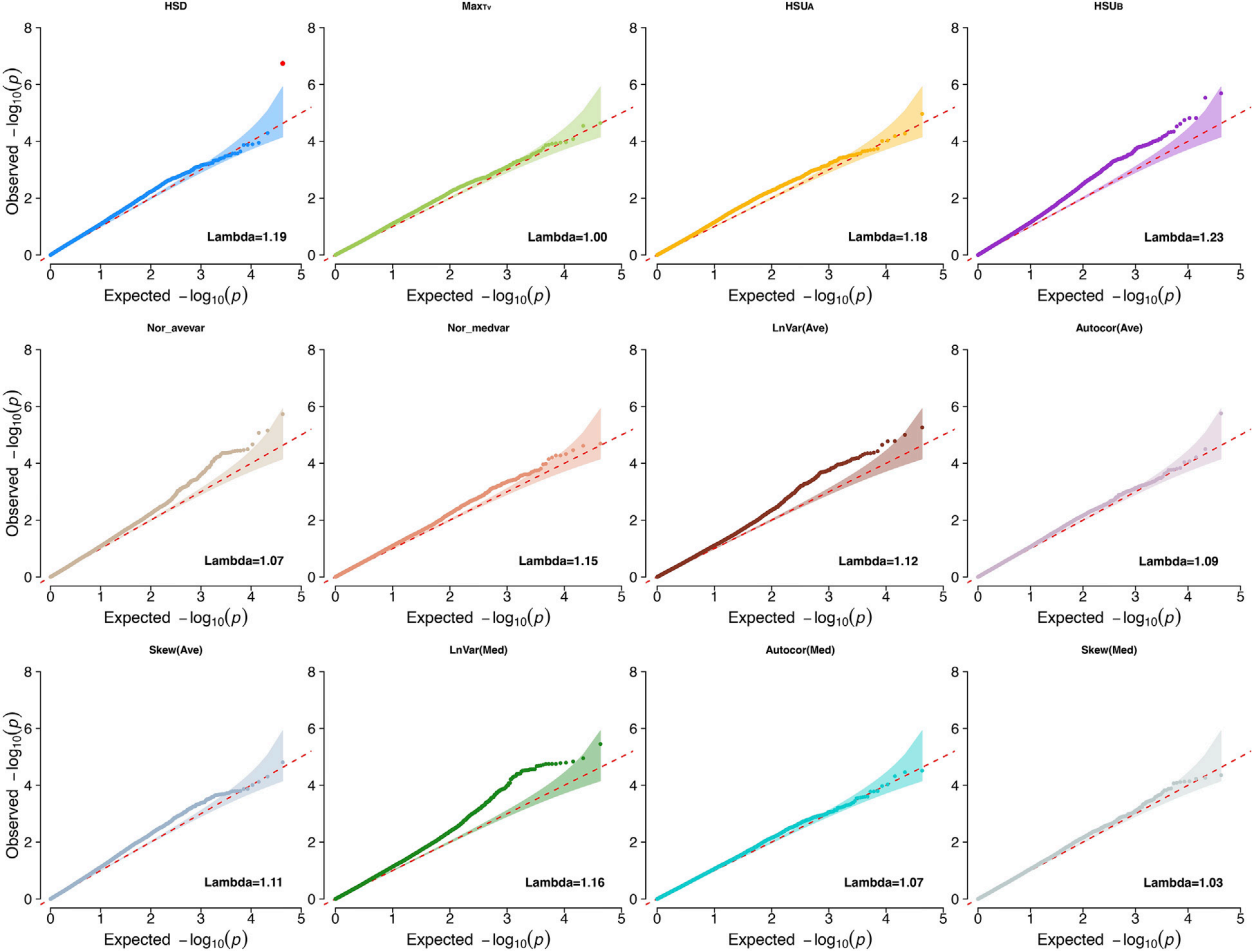
Quantile-quantile plots (QQ-plot) and lambda values for the climatic resilience indicators evaluated^1^. ^1^Indicators: LnVar(Ave), log-transformed variance of the deviations between each observation and the average values from moving windows that contains six continuous observations with 10-mins interval in between; Autocor (Ave): Lag-1 autocorrelation of the deviations between the average values from moving windows that contains six continuous observations with 10-mins interval in between; Skew (Ave): skewness of the deviations between each observation and the average values from moving windows that contains six continuous observations with 10-mins interval in between; LnVar(Med): log-transformed variance of the deviations between the median values from moving windows that contains six continuous observations with 10-mins interval in between; Autocor (Med): Lag-1 autocorrelation of the deviations between the median values from moving windows that contains six continuous observations with 10-mins interval in between; Skew (Med): skewness of the deviations between each observation and the median values from moving windows that contains six continuous observations with 10-mins interval in between; Nor_avevar: normalized average T_V_ multiplies the normalized T_V_ variance; Nor_medvar: normalized median T_V_ multiplies the normalized T_V_ variance; HSU_A_: sum of T_V_ values above the HS threshold during the whole data collection period; HSU_B_: sum of T_V_ values below the HS threshold during the whole data collection period; HSD: The length of time during which the body temperature remained above the HS threshold value for each collection day; Max_Tv_: The highest T_V_ of each measurement day.

We first conducted GWAS studies for all traits to investigate the genetic basis and biological mechanisms associated with heritable CR indicators [heritability estimates ranging from 0.084 ± 0.037 to 0.291 ± 0.047 (Wen et al., 2024)]. The traits evaluated were LnVar(Ave), Autocor(Ave), Skew(Ave), LnVar(Med), Autocor(Med), Skew(Med), Max_Tv_, HSD, Nor_avevar, Nor_medvar, HSU_A_, and HSU_B_. The genomic inflation factors ranged from 0.95 to 1.2 for all indicators, showing small inflation of *P*-values for the estimated SNP effects (Price et al., 2010). Lambda values and Q-Q plots for each CR trait are shown in Figure 1.

Thirty-one SNPs located on nine *Sus scrofa* chromosomes (SSC) that reached at least the suggestive significance level were detected for nine CR indicators and presented in Table 1. Four, one, one, 13, one, four, one, five, one SNP at the suggestive threshold was detected for LnVar(Ave), Autocor (Ave), Skew (Ave), LnVar (Med), Nor_medvar, Nor_avevar, HSU_A_, HSU_B_, and HSD, respectively. Among these SNPs, one SNP located on SSC15:135,366,143 bp, one SNP on SSC6:16,449,770 bp, one SNP on SSC6:16,323,291 bp, and three SNPs on SSC2:88,327,932 bp and 88,631,882 bp and SSC3:18,088,863 bp, were identified as significant at the chromosome-wise level threshold for four CR indicators: Autocor (Ave), LnVar(Med), HSU_B_, and Nor_avevar, respectively. Notably, only one SNP (SSC9: 15,692,376 bp) for HSD met the most stringent significance thresholds (Table 1). No significant associations were found for Max_Tv_, Autocor (Med), and Skew (Med), and this may be lack of power. The small number of suggestive SNPs for each indicator in our study indicates that the evaluated CR indicators are highly polygenic, with many genomic regions of small effects located throughout the different chromosomes. Most HS or heat tolerance related traits in livestock are polygenic (Macciotta et al., 2017; Tiezzi et al., 2020; Cheruiyot et al., 2021), with few major genes identified. Larger sample sizes could be beneficial for identifying these QTLs with smaller effects. These findings are in agreement with our previous research (Freitas et al., 2023; Wen et al., 2023).

**TABLE 1:** Candidate SNPs that reached the suggestive significance level for CR indicators.

SSC^a^	Position	Freq^b^	*P*-value	Indicator	Bonferroni	Chromosome-wise level	Suggestive level
2	8,775,302	0.47	1.08 × 10^−5^	HSU_A_			Significant
6	16,323,291	0.28	2.04 × 10^−6^	HSU_B_		Significant	Significant
6	16,390,288	0.27	2.93 × 10^−6^	HSU_B_			Significant
6	16,449,770	0.14	1.52 × 10^−5^	HSU_B_			Significant
9	15,692,376	0.18	1.52 × 10^−5^	HSU_B_			Significant
9	72,539,075	0.42	1.78 × 10^−5^	HSU_B_			Significant
9	15,692,376	0.18	1.81 × 10^−7^	HSD	Significant	Significant	Significant
1	57,981,015	0.47	5.31 × 10^−5^	Nor_medvar			Significant
3	18,088,863	0.38	1.86 × 10^−6^	Nor_avevar		Significant	Significant
2	88,631,882	0.40	7.03 × 10^−6^	Nor_avevar		Significant	Significant
2	88,327,932	0.19	8.48 × 10^−6^	Nor_avevar		Significant	Significant
2	25,247,173	0.14	2.15 × 10^−5^	Nor_avevar			Significant
7	39,254,889	0.26	5.48 × 10^−6^	LnVar(Ave)			Significant
6	16,449,770	0.14	9.97 × 10^−6^	LnVar(Ave)			Significant
2	100,913,257	0.12	1.65 × 10^−5^	LnVar(Ave)			Significant
3	141,963,034	0.15	1.68 × 10^−5^	LnVar(Ave)			Significant
15	135,366,143	0.14	1.75 × 10^−6^	Autocor (Ave)		Significant	Significant
13	186,420,881	0.41	0.000718	Skew (Ave)			Significant
6	16,449,770	0.14	3.54 × 10^−6^	LnVar(Med)		Significant	Significant
7	39,254,889	0.26	1.12 × 10^−5^	LnVar(Med)			Significant
16	29,568,619	0.28	1.45 × 10^−5^	LnVar(Med)			Significant
16	29,287,377	0.28	1.60 × 10^−5^	LnVar(Med)			Significant
2	91,661,760	0.13	1.65 × 10^−5^	LnVar(Med)			Significant
6	16,435,748	0.16	1.77 × 10^−5^	LnVar(Med)			Significant
16	29,837,397	0.27	1.77 × 10^−5^	LnVar(Med)			Significant
16	29,102,419	0.28	1.78 × 10^−5^	LnVar(Med)			Significant
16	28,947,227	0.27	1.78 × 10^−5^	LnVar(Med)			Significant
16	29,107,832	0.28	1.90 × 10^−5^	LnVar(Med)			Significant
16	29,645,155	0.28	2.01 × 10^−5^	LnVar(Med)			Significant
16	29,513,888	0.28	2.07 × 10^−5^	LnVar(Med)			Significant
16	29,092,396	0.28	2.13 × 10^−5^	LnVar(Med)			Significant

^a^ SSC, *Sus scrofa* chromosome.

^b^ Freq, allele frequency.

Four common SNPs were identified to be associated with more than one CR indicator. The SNPs are located on SSC6:16,449,770 bp and SSC6:16,435,748 bp [HSU_B_, LnVar(Ave), and LnVar(Med)], SSC7:39,254,889 bp [LnVar(Ave) and LnVar(Med)], and SSC9:15,692,376 (HSU_B_ and HSD). The CR indicators created based on similar metrics are highly correlated at the genetic level, such as LnVar(Ave) and LnVar(Med), or HSU_A_ and HSU_B_ (Wen et al., 2024). Interestingly, the SNPs identified for these correlated indicators are not distributed at similar genomic regions. There are several reasons for that: first, as previously mentioned, these indicators are highly polygenic, and we did not find any major gene control in the CR indicators; Second, the candidate SNPs identified by each indicator are in linkage disequilibrium with their causal variants, explaining why overlapping SNPs are still observed across different indicators.

### 3.2 Candidate genes and functional genomic analyses

A total of 442 positional genes harboring or adjacent to the significant SNPs were mapped, including 212 protein-coding genes, 225 non-coding RNAs, and 5 pseudogenes (Supplementary Additional File S1: Supplementary Table S1). Specifically, 22, three, one, 30, one, 91, 45, 18, and one protein-coding genes were identified to be associated with LnVar(Ave), Autocor(Ave), Skew(Ave), LnVar(Med), Nor_medvar, Nor_avevar, HSU_A_, HSU_B_, and HSD, respectively. For LnVar(Ave), the genomic regions around four significant SNPs (SSC2: 100,913,257 bp; SSC3: 141,963,034 bp; SSC6: 16,449,770 bp; SSC7: 39,254,889 bp) harbors Heat Shock Protein 90 Alpha Family Class B Member 1 (*HSP90AB1*), Hyperpolarization Activated Cyclic Nucleotide Gated Potassium Channel 1 (*HCN1*), SPT3 Homolog SAGA And STAGA Complex Component (*SUPT3H*), and Transmembrane Protein 63B (*TMEM63B*) genes. *HSP90AB1* functions as a chaperone and plays a role in protein transport and degradation. Its expression level decreased significantly in response to HS in pigs (Seibert et al., 2019). Besides, one SNP (SNP g.4338T > C) within *HSP90AB1* was found to be significantly related to heat tolerance in Thai indigenous cattle (Charoensook et al., 2012). *HCN1*, *SUPT3H*, and *TMEM63B* were found to be associated with cellular and oxidative stress response in salmon (Beemelmanns et al., 2021), milk production in Holstein cattle (Liu et al., 2021), and residual feed intake in purebred French Large White pigs (Messad et al., 2021), respectively.

One SNP, located at 135,366,143 bp on SSC15, was associated with Autocor (Ave) at chromosome-wise and suggestive significance level. The genomic region around this SNP contains SH3 Domain Binding Protein 4 (*SH3BP4*) and ArfGAP with GTPase Domain, Ankyrin Repeat and PH Domain 1 (*AGAP1*). *SH3BP4* was identified in a region with copy number variation in South African Nguni cattle, which are recognized for their ability to sustain harsh environmental conditions and resistance to parasites and disease (Wang et al., 2015). There were a few peaks with significant SNPs for LnVar(Med) on SSC2: 91,661,760 bp, SSC6: 16.435–16.449 Mb, SSC7: 39,254,889 bp, and between 28.947 and 29.837 Mb on SSC16. The up and downstream of the significant SNPs covered 15 candidate genes that were enriched for LnVar(Ave) before due to the overlapping SNPs. Strong associations were found on SSC2 and SSC3, with Low-Density Lipoprotein Receptor Class A Domain Containing 3 (*LDLRAD3*), solute carrier family 1 member 2 (*SLC1A2*), Dimethylglycine Dehydrogenase (*DMGDH*), Betaine-Homocysteine S-Methyltransferase 2 (*BHMT2*), and Homer Scaffold Protein 1 (*HOMER1*), harboring the most significant SNPs for Nor_avevar. *LDLRAD3*, known to encode a low-density lipoprotein (LDL) receptor and associated with decreased levels of very low-density lipoprotein receptor, has been linked to HS in chickens (Jastrebski et al., 2017; Wang et al., 2020). Further research in mice has revealed that the very low-density lipoprotein receptor plays a crucial role in regulating thermogenesis in brown adipocytes, suggesting its importance in body temperature regulation (Shin et al., 2022). It has been demonstrated that genes encoding the very low-density lipoprotein receptor are crucial for both lipid metabolism and the response to temperature stress (Álvarez et al., 2020). *SLC1A2* was significantly downregulated in the mouse pituitary gland under hot conditions and was related to stress response (Memon et al., 2016). *DMGDH* was considered a candidate gene for heat tolerance, defined as the rate of decline (slope) in milk, fat, and protein yield in swamp buffalos. Furthermore, *DMGDH* may be involved in alleviating oxidative stress in heat-stressed cattle (Cheruiyot et al., 2021). *BHMT2* is involved in regulating homocysteine metabolism with beneficial effects in heat-stressed animals through its activity against osmotic stress and protection of protein denaturation (Cottrell et al., 2015; Del Vesco et al., 2015). Besides, *BHMT2* has been identified as a positively selected candidate gene affecting thermotolerance in African indigenous cattle (Ankole, Ogaden, N'Dama, Boran, and Kenana cattle), using XP-CLR and XP-EHH population statistics (Taye et al., 2017). *HOMER1* plays an important role in behavior, particularly concerning adaptation to stress and fear responses (Kamprath et al., 2009). For Skew(Ave) and Nor_medvar, only one protein-coding gene was identified for each trait–*ENSSSCG00000042482* and *ENSSSCG00000052428*, respectively. However, no information regarding their functions was found for these two genes.

The genes Solute Carrier Family 3 Member 2 (*SLC3A2*), Syntaxin 5 (*STX5*), RNA Polymerase II Subunit G (*POLR2G*), and Glucosidase II Alpha Subunit (*GANAB*) were significantly enriched for HSU_A_. Previous research observed downregulated *SLC3A2* gene expression in bovine mammary epithelial cells under HS conditions (Ma et al., 2018), and this might be an adaptive response to meet increased amino acid requirements during HS (Rhoads et al., 2011). A frameshift mutation in *STX5* has been considered a potential causal mutation for cattle’s heat tolerance, and it also significantly impacts milk production (Cheruiyot et al., 2021). Besides, *STX5* was linked to tick resistance in Belmont Red cattle (Tabor et al., 2017) and Tunisian indigenous sheep (Ahbara et al., 2022). Tick burdens might correlate with thermal comfort (Rocha et al., 2019), as traits such as skin thickness, hair density, and skin secretions influence both tick resistance and heat regulation (Shyma et al., 2015). *GANAB* was found to be downregulated in jejunum mucosa of German Holstein cows under HS conditions, and this is related to responses to incorrect protein folding and stabilization processes (Koch et al., 2021). For HSU_B_, Cytochrome P450 Family 51 Subfamily A Member 1 (*CYP51A1*), and Cyclin Dependent Kinase 6 (*CDK6*) were detected in the SSC9. *CYP51A1*, a gene involved in cholesterol and sterol metabolism, was observed to be upregulated in the plasma of laying hens in response to HS (Zhu et al., 2019). *CDK6* was significantly downregulated by HS in duck granulosa cells (Yang et al., 2021).

Only one protein-coding gene (*ENSSSCG00000034240*) was annotated for the only significant SNP of HSD. The limited overlap between candidate genes identified for various CR indicators in this study and candidate genes from GWAS of resilience for HS is not surprising. First, the traits we used to define resilience, such as HSD and LnVar(Med), differ from those in many other studies (Cheruiyot et al., 2021; Tsartsianidou et al., 2021). Additionally, automatically measured T_V_ enables us to get more accurate CR indicator values. Given the complexity of CR that spans a broad spectrum of adaptive responses, from behavioral to physiological to cellular, it is likely that varying QTLs are captured based on the indicators employed in GWAS studies.

To investigate the biological functions of these candidate genes further, we performed GO and KEGG analysis using DAVID, as shown in Tables 2, 3. Two, one, and one significant KEGG pathways were observed for Lnvar(Med), Lnvar(Ave), and Nor_avevar, respectively. These pathways are related to stress response [e.g., chemical carcinogenesis - receptor activation (Trush and Kensler, 1991)], immune and inflammatory responses [e.g., Th17 cell differentiation (Guo et al., 2018)], cell survival, proliferation, and migration [e.g., PI3K/Akt signaling pathway (Zhang et al., 2016; Yiming et al., 2021)], and nervous system (e.g., Glutamatergic synapse) (Niciu et al., 2012). Heat stress also has been documented to cause a change in animals’ adaptive immune function, transitioning from the typical cell-mediated to humoral immunity (Niciu et al., 2012). This shift can subsequently result in a weakened immune system, making the animal more susceptible to numerous pathogens (Calapre et al., 2013a). Heat stress has been shown to activate heat shock proteins (HSPs), which can promote cell proliferation and survival (Srikanth et al., 2017). Research found that HSPs are overexpressed in various cancers and have been implicated in carcinogenesis (Ciocca and Calderwood, 2005). Heat stress has also been documented to cause a change in animals’ adaptive immune function, transitioning from the typical cell-mediated to humoral immunity (Sophia et al., 2016). This shift can subsequently result in a weakened immune system, making the animal more susceptible to numerous pathogens (Vandana et al., 2019). Additionally, HS can lead to oxidative stress in various livestock, such as dairy cattle (Bernabucci et al., 2002), pigs (Liu et al., 2016), sheep (Chauhan et al., 2016), and poultry (Shakeri et al., 2019). This heightens their vulnerability to numerous pathogens and production-related illnesses.

**TABLE 2:** Significantly enriched (*P* < 0.05) Gene Ontology (GO) terms identified for CR indicators.

Indicator^a^	Category^b^	Term	*P*-value
LnVar(Ave)	BP	GO:0031334, positive regulation of protein complex assembly	0.03
	BP	GO:0033138, positive regulation of peptidyl-serine phosphorylation	0.07
LnVar(Med)	BP	GO:0007155, cell adhesion	0.00
	BP	GO:0050918, positive chemotaxis	0.01
	BP	GO:0050930, induction of positive chemotaxis	0.01
	BP	GO:0006310, DNA recombination	0.02
	BP	GO:0006302, double-strand break repair	0.02
	BP	GO:0050731, positive regulation of peptidyl-tyrosine phosphorylation	0.02
	BP	GO:0001501, skeletal system development	0.03
	BP	GO:0007417, central nervous system development	0.03
	BP	GO:0033138, positive regulation of peptidyl-serine phosphorylation	0.04
	MF	GO:0005540, hyaluronic acid binding	0.00
	MF	GO:0042056, chemoattractant activity	0.02
	MF	GO:0005509, calcium ion binding	0.03
	CC	GO:0005576, extracellular region	0.06
Nor_avevar	BP	GO:0009086, methionine biosynthetic process	0.01
	BP	GO:0031401, positive regulation of protein modification process	0.02
	BP	GO:0051403, stress-activated MAPK cascade	0.02
	BP	GO:0070613, regulation of protein processing	0.03
	BP	GO:0010762, regulation of fibroblast migration	0.03
	BP	GO:0006357, regulation of transcription from RNA polymerase II promoter	0.03
	MF	GO:0047150, betaine-homocysteine S-methyltransferase activity	0.01
	CC	GO:0005829, cytosol	0.00
	CC	GO:0005769, early endosome	0.02
	CC	GO:0008021, synaptic vesicle	0.03
	CC	GO:0098978, glutamatergic synapse	0.04
HSU_A_	BP	GO:0015711, organic anion transport	0.00
	BP	GO:0015732, prostaglandin transport	0.01
	BP	GO:0005975, carbohydrate metabolic process	0.02
	MF	GO:0022857, transmembrane transporter activity	0.00
	CC	GO:0016323, basolateral plasma membrane	0.02
	CC	GO:0005654, nucleoplasm	0.03

^a^ Indicators: LnVar(Ave): log-transformed variance of the deviations between each observation and the average values from moving windows that contains six continuous observations with 10-mins interval in between; LnVar(Med), log-transformed variance of the deviations between the median values from moving windows that contains six continuous observations with 10-mins interval in between; Nor_avevar, normalized average T_V_, multiplies the normalized T_V_, variance; HSU_A_: sum of Tv values above the HS, threshold during the whole data collection period.

^b^ Category: BP, biological process; MF, molecular function; CC, cellular component.

**TABLE 3:** Significantly enriched (*P* < 0.05) Kyoto Encyclopedia of Genes and Genomes (KEGG) pathways identified for CR indicators.

Indicator^a^	Term	P-Value
LnVar(Ave)	ssc04659, Th17 cell differentiation	0.02
LnVar(Med)	ssc05207, Chemical carcinogenesis - receptor activation	0.02
	ssc04151, PI3K-Akt signaling pathway	0.03
Nor_avevar	ssc04724, Glutamatergic synapse	0.04

^a^ Indicators: LnVar (Ave), log-transformed variance of the deviations between each observation and the average values from moving windows that contains six continuous observations with 10-mins interval in between; LnVar (Med), log-transformed variance of the deviations between the median values from moving windows that contains six continuous observations with 10-mins interval in between; Nor_avevar, normalized average T_V_, multiplies the normalized T_V_, variance.

In addition, studies have shown that the immune responses in organisms are extremely sensitive to DNA damage that is caused by stressors (Nakad and Schumacher, 2016). The PI3K/AKT signaling pathway is involved in intracellular responses by reactive oxygen species (ROS) and inflammation caused by DNA fragmentation (Datta et al., 2023). Heat stress-induced testicular damage could be alleviated with melatonin, a potent antioxidant, in dairy goats by inhibiting the PI3K/AKT signaling pathway (Liu et al., 2022). Previous research has indicated that thermal stress leads to a reduction in glutamatergic synapse transmission (Popoli et al., 2012). Besides, glutamatergic synapses have been demonstrated to play roles in HSPs synthesis. This synthesis aids in repairing stress-induced synaptic protein damage and bolsters neuroprotective mechanisms (Kiang and Tsokos, 1998). Heat tolerance, defined as the rate of decline in milk production (slope traits) in response to a rising temperature–humidity index, is significantly associated with the enrichment of the glutamatergic synapse pathway in Holstein cows (Cheruiyot et al., 2021).

A total of 13, two, 17, eight, and one significant GO terms were enriched for Lnvar(Med), Lnvar(Ave), Nor_avevar, HSU_A_, and HSU_B_, respectively. The functions of enriched GO terms are similar to those of KEGG pathways. These GO terms are related to DNA damage and repair (e.g., DNA recombination, double-strand break repair), stress responses (e.g., stress-activated MAPK cascade), protein modifications (e.g., positive regulation of peptidyl-serine phosphorylation, positive regulation of peptidyl-tyrosine phosphorylation, and positive regulation of protein complex assembly), nervous system (e.g., central nervous system development, glutamatergic synapse, and synaptic vesicle), and cell structure and mechanics. Various types of DNA damage, including the induction of double-strand breaks in DNA (Ning et al., 2021; Habibi et al., 2022), are directly affected by HS. DNA recombination is one mechanism cells use to repair certain types of DNA damage. Previous research showed that the upregulated genes are mainly involved in DNA or protein damage/recombination, cell cycle processes, biogenesis, and stress and immune responses using transcriptome analysis in heat-stressed finishing pigs (Ma et al., 2019).

A substantial number of phosphorylation changes are induced by severe heat stress and occur with kinetics similar to the inhibition of protein synthesis (Duncan and Hershey, 1989). This has been evidenced by the detection of phosphorylation-related GO terms and functions in various species under HS conditions, including buffalos (Muthukumar et al., 2014), broilers (Kim et al., 2022), and swine (Yu et al., 2010; Cross et al., 2018). Notably, phosphorylation is crucial for the transcriptional activity of the heat shock transcription factor 1 and for triggering the heat shock response (Holmberg et al., 2001). Moreover, HS has been shown to activate MAPK phosphorylation in different cell types, such as intestinal cells, lung fibroblasts, and chondrocytes (Liu et al., 2019), and it has also been associated with cell and tissue injury (Banerjee Mustafi et al., 2009).

The genomic regions around candidate SNPs are shown to be linked with QTL regions associated with different traits. The major fraction of QTL annotated in this study belonged to the “Meat and Carcass” type, which accounts for 43.75% of the total QTL, and average daily gain and bone weight were the most traits that we enriched for Meat and Carcass QTL. Meanwhile, the genomic regions identified overlapped with several QTLs previously related to production, reproduction, health, and exterior traits (conformation and appearance), as shown in Table 4.

**TABLE 4:** Significantly enriched (*P* < 0.05) QTL identified for CR indicators.

Indicator^a^	SSC^b^	QTL type^c^	Trait
LnVar(Ave)	6	Meat_and_Carcass	Bone weight
	6	Exterior	Maternal infanticide
	7	Production	Body weight
	7	Production	Average daily gain
	7	Production	Average daily gain
	7	Meat_and_Carcass	Skin weight
Skew (Ave)	13	Meat_and_Carcass	Acid flavor
HSU_A_	2	Meat_and_Carcass	Muscle conductivity
	2	Meat_and_Carcass	Average chain length
	2	Reproduction	Number of stillborn
	2	Reproduction	uterine horn weight
HSU_B_	6	Meat_and_Carcass	Bone weight
	6	Exterior	Maternal infanticide
	6	Meat_and_Carcass	Bone weight
	6	Exterior	Maternal infanticide
	9	Meat_and_Carcass	Carcass length
Nor_avevar	2	Health	Insulin-like growth factor 1 level
	2	Health	Hemolytic complement activity (classical pathway)
	2	Meat_and_Carcass	Shear force
	2	Meat_and_Carcass	tenderness score
	2	Reproduction	Teat number
	3	Meat_and_Carcass	Fat androstenone level
LnVar(Med)	2	Meat_and_Carcass	Shear force
	6	Meat_and_Carcass	Bone weight
	6	Exterior	Maternal infanticide
	7	Production	Body weight
	7	Production	Average daily gain
	7	Production	Average daily gain
	7	Meat_and_Carcass	Skin weight
	16	Reproduction	Teat number
	16	Exterior	Lumbar vertebra number
	16	Exterior	Lumbar vertebra number

^a^ Indicators: LnVar(Ave), log-transformed variance of the deviations between each observation and the average values from moving windows that contains six continuous observations with 10-mins interval in between; Skew (Ave), skewness of the deviations between each observation and the average values from moving windows that contains six continuous observations with 10-mins interval in between; LnVar(Med), log-transformed variance of the deviations between the median values from moving windows that contains six continuous observations with 10-mins interval in between; Nor_avevar, normalized average T_V_, multiplies the normalized T_V_, variance; HSU_A_: sum of Tv values above the HS, threshold during the whole data collection period; HSU_B_: sum of Tv values below the HS, threshold during the whole data collection period.

^b^ SSC, *sus scrofa* chromosome.

^c^ QTL, type: main type of QTL, trait group previously identified.

This is the first GWAS for CR indicators derived from automatically measured T_V_. These significant SNPs hold great potential for enhancing genomic predictions for CR in pigs, by incorporating more SNPs located in the regions of these significant SNPs into existing commercial SNP panels to improve the prediction accuracy. Small sample size may limited the power of analysis, this analysis should be conducted in a larger population. Besides, different weights for these important SNPs or genes could be given through biology-driven genomic prediction methods [e.g., different subsets of SNPs were used for genomic predictions (Li et al., 2018)]. However, even though using different significance thresholds for GWAS, identifying the causal mutations for these CR indicators remains challenging due to the linkage disequilibrium. It would be better to use whole genome sequencing data to fully capture LD patterns, thereby achieving higher GWAS power compared to array-based GWAS (Pengelly et al., 2015). Thus, future research efforts should prioritize additional biological validations. In our study, we used a crossbred population. Similar analyses should be conducted in other populations with different genetic backgrounds to determine if the CR indicators are universally applicable.

## 4 Conclusion

The study focuses on sows and explores various CR indicators to better understand the genetic factors and biological mechanisms behind climatic resilience. We identified multiple genetic regions associated with different CR indicators, revealing that CR is a highly polygenic trait with small effect sizes distributed across the genome. Furthermore, many heat tolerance or HS related genes in our study, such as *HSP90AB1*, *DMGDH*, and *HOMER1*, have been identified. Additionally, the functional analyses showed the complexity of CR, involving various adaptive responses, from behavioral to cellular. These findings highlight the possibility of selecting more heat-tolerant individuals based on the identified SNP for CR indicators.

## Data Availability

The data analyzed in this study is subject to the following licenses/restrictions: The phenotypic and genomic data used in this study are a property of the industry partner that contributed to the study and therefore are not readily available due to its commercially sensitivity. Requests to access these datasets should be directed to the corresponding author (LB, britol@purdue.edu).
