# Supplementary material for: Genomic regions and biological mechanisms underlying climatic resilience traits derived from automatically-recorded vaginal temperature in lactating sows under heat stress conditions
Source: Front Genet. 2024 Nov 7;15:1498380. doi: 10.3389/fgene.2024.1498380 (PMC11578969; doi:10.3389/fgene.2024.1498380)
Supplement: Supplementary file 1 [file Table1.docx]

Supplementary Material

**Supplementary table 1.** Significantly enriched (P < 0.05) genes identified for CR indicators.

| **Indicator** | **ensembl_gene_id** | **entrezgene_id** | **SSC^1^** | **external_gene** | **gene_biotype** |
| --- | --- | --- | --- | --- | --- |
| **LnVar(Ave)** | ENSSSCG00000051601 |  | 2 |  | lncRNA |
|  | ENSSSCG00000061218 |  | 2 |  | lncRNA |
|  | ENSSSCG00000014157 | 100621341 | 2 | NR2F1 | protein_coding |
|  | ENSSSCG00000027608 | 100521436 | 2 | FAM172A | protein_coding |
|  | ENSSSCG00000014161 | 106509502 | 2 | KIAA0825 | protein_coding |
|  | ENSSSCG00000062599 |  | 2 |  | protein_coding |
|  | ENSSSCG00000014163 | 100522125 | 2 | SLF1 | protein_coding |
|  | ENSSSCG00000025286 | 100522480 | 2 | MCTP1 | protein_coding |
|  | ENSSSCG00000053114 |  | 6 |  | lncRNA |
|  | ENSSSCG00000057921 |  | 6 |  | lncRNA |
|  | ENSSSCG00000048469 |  | 6 |  | protein_coding |
|  | ENSSSCG00000047359 |  | 6 |  | lncRNA |
|  | ENSSSCG00000041519 |  | 6 |  | lncRNA |
|  | ENSSSCG00000041474 |  | 6 |  | lncRNA |
|  | ENSSSCG00000056956 |  | 6 |  | lncRNA |
|  | ENSSSCG00000061838 |  | 6 | U6 | snRNA |
|  | ENSSSCG00000063114 |  | 6 |  | lncRNA |
|  | ENSSSCG00000059764 |  | 6 |  | lncRNA |
|  | ENSSSCG00000053257 |  | 6 |  | lncRNA |
|  | ENSSSCG00000061174 |  | 6 |  | lncRNA |
|  | ENSSSCG00000055852 |  | 6 |  | lncRNA |
|  | ENSSSCG00000052407 |  | 6 |  | lncRNA |
|  | ENSSSCG00000048283 |  | 6 |  | lncRNA |
|  | ENSSSCG00000053189 |  | 6 |  | lncRNA |
|  | ENSSSCG00000060193 |  | 6 |  | lncRNA |
|  | ENSSSCG00000054484 |  | 6 |  | lncRNA |
|  | ENSSSCG00000058326 |  | 6 |  | lncRNA |
|  | ENSSSCG00000001695 | 397157 | 7 | VEGFA | protein_coding |
|  | ENSSSCG00000057650 |  | 7 |  | lncRNA |
|  | ENSSSCG00000048551 |  | 7 |  | lncRNA |
|  | ENSSSCG00000063238 |  | 7 |  | lncRNA |
|  | ENSSSCG00000001696 | 100157118 | 7 |  | protein_coding |
|  | ENSSSCG00000001697 | 100151872 | 7 | TMEM63B | protein_coding |
|  | ENSSSCG00000001698 | 100156753 | 7 | CAPN11 | protein_coding |
|  | ENSSSCG00000053910 | 110261530 | 7 | MYMX | protein_coding |
|  | ENSSSCG00000001700 | 396631 | 7 | SLC29A1 | protein_coding |
|  | ENSSSCG00000001701 | 396742 | 7 | HSP90AB1 | protein_coding |
|  | ENSSSCG00000001702 | 100516086 | 7 | SLC35B2 | protein_coding |
|  | ENSSSCG00000001703 | 100153486 | 7 | NFKBIE | protein_coding |
|  | ENSSSCG00000040047 | 100157535 | 7 | TMEM151B | protein_coding |
|  | ENSSSCG00000001705 | 100156326 | 7 | TCTE1 | protein_coding |
|  | ENSSSCG00000001706 | 100155115 | 7 | AARS2 | protein_coding |
|  | ENSSSCG00000041884 |  | 7 |  | lncRNA |
|  | ENSSSCG00000039903 | 100153102 | 7 | SPATS1 | protein_coding |
|  | ENSSSCG00000001708 | 100153900 | 7 | CDC5L | protein_coding |
|  | ENSSSCG00000059458 |  | 7 |  | lncRNA |
|  | ENSSSCG00000057783 |  | 7 |  | lncRNA |
|  | ENSSSCG00000058808 |  | 7 |  | lncRNA |
|  | ENSSSCG00000001709 | 100154712 | 7 | SUPT3H | protein_coding |
| **Autocor(Ave)** | ENSSSCG00000054647 |  | 15 |  | protein_coding |
|  | ENSSSCG00000034692 | 110256943 | 15 | SH3BP4 | protein_coding |
|  | ENSSSCG00000016317 | 100620812 | 15 | AGAP1 | protein_coding |
| **Skew(Ave)** | ENSSSCG00000058611 |  | 13 |  | lncRNA |
|  | ENSSSCG00000034750 |  | 13 |  | processed_pseudogene |
|  | ENSSSCG00000062082 |  | 13 |  | lncRNA |
|  | ENSSSCG00000054473 |  | 13 |  | lncRNA |
|  | ENSSSCG00000019813 |  | 13 | U6 | snRNA |
|  | ENSSSCG00000058163 |  | 13 |  | lncRNA |
|  | ENSSSCG00000055801 |  | 13 |  | lncRNA |
|  | ENSSSCG00000047451 |  | 13 |  | lncRNA |
|  | ENSSSCG00000044410 |  | 13 |  | lncRNA |
|  | ENSSSCG00000056349 |  | 13 |  | lncRNA |
|  | ENSSSCG00000054368 |  | 13 |  | lncRNA |
|  | ENSSSCG00000059213 |  | 13 |  | lncRNA |
|  | ENSSSCG00000052007 |  | 13 |  | lncRNA |
|  | ENSSSCG00000055882 |  | 13 |  | lncRNA |
|  | ENSSSCG00000042482 |  | 13 |  | protein_coding |
|  | ENSSSCG00000061260 |  | 13 |  | lncRNA |
|  | ENSSSCG00000053379 |  | 13 |  | lncRNA |
|  | ENSSSCG00000054272 |  | 13 |  | lncRNA |
|  | ENSSSCG00000056815 |  | 13 |  | lncRNA |
|  | ENSSSCG00000054044 |  | 13 |  | lncRNA |
|  | ENSSSCG00000046211 |  | 13 |  | lncRNA |
|  | ENSSSCG00000059674 |  | 13 |  | lncRNA |
| **LnVar(Med)** | ENSSSCG00000055815 |  | 16 |  | protein_coding |
|  | ENSSSCG00000053699 |  | 16 |  | lncRNA |
|  | ENSSSCG00000047655 |  | 16 |  | lncRNA |
|  | ENSSSCG00000058414 |  | 16 |  | lncRNA |
|  | ENSSSCG00000047302 |  | 16 |  | lncRNA |
|  | ENSSSCG00000016878 | 100525086 | 16 | FGF10 | protein_coding |
|  | ENSSSCG00000061143 |  | 16 |  | lncRNA |
|  | ENSSSCG00000062213 |  | 16 |  | lncRNA |
|  | ENSSSCG00000051828 |  | 16 |  | lncRNA |
|  | ENSSSCG00000046761 |  | 16 |  | lncRNA |
|  | ENSSSCG00000058926 |  | 16 |  | lncRNA |
|  | ENSSSCG00000053324 |  | 16 |  | lncRNA |
|  | ENSSSCG00000053958 |  | 16 |  | lncRNA |
|  | ENSSSCG00000055856 |  | 16 |  | lncRNA |
|  | ENSSSCG00000053561 |  | 16 |  | lncRNA |
|  | ENSSSCG00000032275 | 100525262 | 16 | MRPS30 | protein_coding |
|  | ENSSSCG00000048222 |  | 16 |  | lncRNA |
|  | ENSSSCG00000062385 |  | 16 |  | lncRNA |
|  | ENSSSCG00000043312 |  | 16 |  | lncRNA |
|  | ENSSSCG00000046701 |  | 16 |  | lncRNA |
|  | ENSSSCG00000060450 |  | 16 |  | lncRNA |
|  | ENSSSCG00000060791 |  | 16 |  | lncRNA |
|  | ENSSSCG00000026527 | 100510937 | 16 | HCN1 | protein_coding |
|  | ENSSSCG00000016881 |  | 16 |  | protein_coding |
|  | ENSSSCG00000054542 |  | 16 |  | lncRNA |
|  | ENSSSCG00000043266 |  | 16 |  | processed_pseudogene |
|  | ENSSSCG00000029484 | 100622067 | 16 | EMB | protein_coding |
|  | ENSSSCG00000022691 |  | 16 | SNORD28 | snoRNA |
|  | ENSSSCG00000055818 |  | 16 |  | lncRNA |
|  | ENSSSCG00000016882 | 100511108 | 16 | PARP8 | protein_coding |
|  | ENSSSCG00000060876 |  | 2 |  | lncRNA |
|  | ENSSSCG00000030562 |  | 2 | U6 | snRNA |
|  | ENSSSCG00000056442 | 100514704 | 2 | TMEM167A | protein_coding |
|  | ENSSSCG00000018205 |  | 2 | SCARNA18 | snoRNA |
|  | ENSSSCG00000045510 |  | 2 |  | protein_coding |
|  | ENSSSCG00000014136 | 397328 | 2 | VCAN | protein_coding |
|  | ENSSSCG00000014136 | 100514895 | 2 | VCAN | protein_coding |
|  | ENSSSCG00000038060 |  | 2 |  | protein_coding |
|  | ENSSSCG00000057778 |  | 2 |  | lncRNA |
|  | ENSSSCG00000014137 | 445513 | 2 | HAPLN1 | protein_coding |
|  | ENSSSCG00000063548 |  | 2 |  | lncRNA |
|  | ENSSSCG00000026780 | 100516065 | 2 | EDIL3 | protein_coding |
|  | ENSSSCG00000053114 |  | 6 |  | lncRNA |
|  | ENSSSCG00000057921 |  | 6 |  | lncRNA |
|  | ENSSSCG00000048469 |  | 6 |  | protein_coding |
|  | ENSSSCG00000047359 |  | 6 |  | lncRNA |
|  | ENSSSCG00000041519 |  | 6 |  | lncRNA |
|  | ENSSSCG00000041474 |  | 6 |  | lncRNA |
|  | ENSSSCG00000056956 |  | 6 |  | lncRNA |
|  | ENSSSCG00000061838 |  | 6 | U6 | snRNA |
|  | ENSSSCG00000063114 |  | 6 |  | lncRNA |
|  | ENSSSCG00000059764 |  | 6 |  | lncRNA |
|  | ENSSSCG00000053257 |  | 6 |  | lncRNA |
|  | ENSSSCG00000061174 |  | 6 |  | lncRNA |
|  | ENSSSCG00000055852 |  | 6 |  | lncRNA |
|  | ENSSSCG00000052407 |  | 6 |  | lncRNA |
|  | ENSSSCG00000048283 |  | 6 |  | lncRNA |
|  | ENSSSCG00000053189 |  | 6 |  | lncRNA |
|  | ENSSSCG00000060193 |  | 6 |  | lncRNA |
|  | ENSSSCG00000054484 |  | 6 |  | lncRNA |
|  | ENSSSCG00000058326 |  | 6 |  | lncRNA |
|  | ENSSSCG00000001695 | 397157 | 7 | VEGFA | protein_coding |
|  | ENSSSCG00000057650 |  | 7 |  | lncRNA |
|  | ENSSSCG00000048551 |  | 7 |  | lncRNA |
|  | ENSSSCG00000063238 |  | 7 |  | lncRNA |
|  | ENSSSCG00000001696 | 100157118 | 7 |  | protein_coding |
|  | ENSSSCG00000001697 | 100151872 | 7 | TMEM63B | protein_coding |
|  | ENSSSCG00000001698 | 100156753 | 7 | CAPN11 | protein_coding |
|  | ENSSSCG00000053910 | 110261530 | 7 | MYMX | protein_coding |
|  | ENSSSCG00000001700 | 396631 | 7 | SLC29A1 | protein_coding |
|  | ENSSSCG00000001701 | 396742 | 7 | HSP90AB1 | protein_coding |
|  | ENSSSCG00000001702 | 100516086 | 7 | SLC35B2 | protein_coding |
|  | ENSSSCG00000001703 | 100153486 | 7 | NFKBIE | protein_coding |
|  | ENSSSCG00000040047 | 100157535 | 7 | TMEM151B | protein_coding |
|  | ENSSSCG00000001705 | 100156326 | 7 | TCTE1 | protein_coding |
|  | ENSSSCG00000001706 | 100155115 | 7 | AARS2 | protein_coding |
|  | ENSSSCG00000041884 |  | 7 |  | lncRNA |
|  | ENSSSCG00000039903 | 100153102 | 7 | SPATS1 | protein_coding |
|  | ENSSSCG00000001708 | 100153900 | 7 | CDC5L | protein_coding |
|  | ENSSSCG00000059458 |  | 7 |  | lncRNA |
|  | ENSSSCG00000057783 |  | 7 |  | lncRNA |
|  | ENSSSCG00000058808 |  | 7 |  | lncRNA |
|  | ENSSSCG00000001709 | 100154712 | 7 | SUPT3H | protein_coding |
| **Nor_MedVar** | ENSSSCG00000055071 |  | 1 |  | lncRNA |
|  | ENSSSCG00000061045 |  | 1 |  | lncRNA |
|  | ENSSSCG00000058530 |  | 1 |  | lncRNA |
|  | ENSSSCG00000062584 |  | 1 |  | lncRNA |
|  | ENSSSCG00000042598 |  | 1 |  | lncRNA |
|  | ENSSSCG00000054767 |  | 1 |  | lncRNA |
|  | ENSSSCG00000052428 |  | 1 |  | protein_coding |
|  | ENSSSCG00000045802 |  | 1 |  | processed_pseudogene |
|  | ENSSSCG00000059105 |  | 1 |  | lncRNA |
| **Nor_AveVar** | ENSSSCG00000013292 | 100523837 | 2 | PRR5L | protein_coding |
|  | ENSSSCG00000046177 |  | 2 |  | protein_coding |
|  | ENSSSCG00000013293 | 100524189 | 2 | COMMD9 | protein_coding |
|  | ENSSSCG00000046114 |  | 2 |  | protein_coding |
|  | ENSSSCG00000048614 |  | 2 |  | lncRNA |
|  | ENSSSCG00000013294 | 100511916 | 2 | LDLRAD3 | protein_coding |
|  | ENSSSCG00000062228 |  | 2 |  | protein_coding |
|  | ENSSSCG00000047141 |  | 2 |  | lncRNA |
|  | ENSSSCG00000054951 |  | 2 |  | lncRNA |
|  | ENSSSCG00000049955 |  | 2 |  | lncRNA |
|  | ENSSSCG00000061822 |  | 2 |  | lncRNA |
|  | ENSSSCG00000057682 |  | 2 |  | lncRNA |
|  | ENSSSCG00000054809 |  | 2 |  | lncRNA |
|  | ENSSSCG00000013295 | 100524974 | 2 | TRIM44 | protein_coding |
|  | ENSSSCG00000025588 | 100626682 | 2 | FJX1 | protein_coding |
|  | ENSSSCG00000040861 |  | 2 |  | protein_coding |
|  | ENSSSCG00000060910 |  | 2 |  | lncRNA |
|  | ENSSSCG00000052582 |  | 2 |  | lncRNA |
|  | ENSSSCG00000028338 | 100626770 | 2 | PAMR1 | protein_coding |
|  | ENSSSCG00000060266 |  | 2 |  | lncRNA |
|  | ENSSSCG00000013296 | 100627068 | 2 | SLC1A2 | protein_coding |
|  | ENSSSCG00000014110 | 100525563 | 2 | DMGDH | protein_coding |
|  | ENSSSCG00000054426 | 100523776 | 2 | BHMT2 | protein_coding |
|  | ENSSSCG00000053764 |  | 2 |  | protein_coding |
|  | ENSSSCG00000052026 |  | 2 |  | lncRNA |
|  | ENSSSCG00000058054 |  | 2 |  | lncRNA |
|  | ENSSSCG00000014108 | 397371 | 2 | BHMT | protein_coding |
|  | ENSSSCG00000014112 | 100510894 | 2 | JMY | protein_coding |
|  | ENSSSCG00000054089 |  | 2 |  | lncRNA |
|  | ENSSSCG00000014113 | 100511070 | 2 | HOMER1 | protein_coding |
|  | ENSSSCG00000043942 |  | 2 |  | protein_coding |
|  | ENSSSCG00000014114 | 100512336 | 2 | TENT2 | protein_coding |
|  | ENSSSCG00000026098 | 100505410 | 2 | CMYA5 | protein_coding |
|  | ENSSSCG00000059163 |  | 2 |  | lncRNA |
|  | ENSSSCG00000014116 | 100512828 | 2 | MTX3 | protein_coding |
|  | ENSSSCG00000014117 | 100513005 | 2 | THBS4 | protein_coding |
|  | ENSSSCG00000014119 | 100624743 | 2 | SERINC5 | protein_coding |
|  | ENSSSCG00000019576 |  | 2 | U1 | snRNA |
|  | ENSSSCG00000014122 | 100624840 | 2 | SPZ1 | protein_coding |
|  | ENSSSCG00000014121 | 100511806 | 2 | ZFYVE16 | protein_coding |
|  | ENSSSCG00000014123 | 100511985 | 2 | FAM151B | protein_coding |
|  | ENSSSCG00000041587 |  | 3 |  | lncRNA |
|  | ENSSSCG00000007780 | 100513588 | 3 | ZNF629 | protein_coding |
|  | ENSSSCG00000058184 |  | 3 |  | protein_coding |
|  | ENSSSCG00000007786 | 100515077 | 3 | RNF40 | protein_coding |
|  | ENSSSCG00000007778 | 100513786 | 3 | CFAP119 | protein_coding |
|  | ENSSSCG00000007788 | 100310801 | 3 | PHKG2 | protein_coding |
|  | ENSSSCG00000058422 | 100513980 | 3 |  | protein_coding |
|  | ENSSSCG00000007789 | 100514159 | 3 | SRCAP | protein_coding |
|  | ENSSSCG00000019607 |  | 3 | SNORA30 | snoRNA |
|  | ENSSSCG00000025794 | 102159616 | 3 | FBRS | protein_coding |
|  | ENSSSCG00000048870 |  | 3 |  | protein_coding |
|  | ENSSSCG00000007790 | 100514519 | 3 | PRR14 | protein_coding |
|  | ENSSSCG00000047341 |  | 3 |  | protein_coding |
|  | ENSSSCG00000033496 | 106508768 | 3 | ZNF689 | protein_coding |
|  | ENSSSCG00000037875 | 100515603 | 3 | ZNF688 | protein_coding |
|  | ENSSSCG00000038852 | 100620532 | 3 |  | protein_coding |
|  | ENSSSCG00000023349 | 100620993 | 3 | ZNF768 | protein_coding |
|  | ENSSSCG00000007797 | 733681 | 3 | ITGAL | protein_coding |
|  | ENSSSCG00000007795 | 100621901 | 3 | DCTPP1 | protein_coding |
|  | ENSSSCG00000040745 | 100621101 | 3 | ZNF771 | protein_coding |
|  | ENSSSCG00000060090 |  | 3 |  | lncRNA |
|  | ENSSSCG00000007801 | 100621309 | 3 | ZNF48 | protein_coding |
|  | ENSSSCG00000007800 | 100514708 | 3 | SEPTIN1 | protein_coding |
|  | ENSSSCG00000007799 | 474162 | 3 | MYL11 | protein_coding |
|  | ENSSSCG00000007798 | 100514343 | 3 | TBC1D10B | protein_coding |
|  | ENSSSCG00000007791 | 100621208 | 3 | CD2BP2 | protein_coding |
|  | ENSSSCG00000035256 | 100623653 | 3 | SPN | protein_coding |
|  | ENSSSCG00000027454 | 100623339 | 3 | QPRT | protein_coding |
|  | ENSSSCG00000029752 | 100623246 | 3 |  | protein_coding |
|  | ENSSSCG00000029509 | 100623146 | 3 | KIF22 | protein_coding |
|  | ENSSSCG00000025321 | 100622994 | 3 | MAZ | protein_coding |
|  | ENSSSCG00000027970 | 100623439 | 3 | PRRT2 | protein_coding |
|  | ENSSSCG00000054721 | 100622905 | 3 | PAGR1 | protein_coding |
|  | ENSSSCG00000027946 | 100622738 | 3 | MVP | protein_coding |
|  | ENSSSCG00000027629 | 100312963 | 3 | CDIPT | protein_coding |
|  | ENSSSCG00000034445 | 100622642 | 3 |  | protein_coding |
|  | ENSSSCG00000029186 | 100622262 | 3 | SEZ6L2 | protein_coding |
|  | ENSSSCG00000040342 | 100623843 | 3 |  | protein_coding |
|  | ENSSSCG00000040888 | 100623749 | 3 | KCTD13 | protein_coding |
|  | ENSSSCG00000052107 | 100623961 | 3 | TMEM219 | protein_coding |
|  | ENSSSCG00000031967 | 100624059 | 3 | TAOK2 | protein_coding |
|  | ENSSSCG00000030122 | 100624465 | 3 | HIRIP3 | protein_coding |
|  | ENSSSCG00000032921 | 100624556 | 3 | INO80E | protein_coding |
|  | ENSSSCG00000034635 | 100624654 | 3 | DOC2A | protein_coding |
|  | ENSSSCG00000037075 |  | 3 | C16orf92 | protein_coding |
|  | ENSSSCG00000037193 | 100624742 | 3 | TLCD3B | protein_coding |
|  | ENSSSCG00000046707 |  | 3 |  | protein_coding |
|  | ENSSSCG00000032556 | 110260081 | 3 | ALDOA | protein_coding |
|  | ENSSSCG00000036817 | 110260085 | 3 | PPP4C | protein_coding |
|  | ENSSSCG00000034774 | 110260084 | 3 | TBX6 | protein_coding |
|  | ENSSSCG00000035430 | 100624297 | 3 | YPEL3 | protein_coding |
|  | ENSSSCG00000021811 | 100620986 | 3 | GDPD3 | protein_coding |
|  | ENSSSCG00000040682 | 445013 | 3 | MAPK3 | protein_coding |
|  | ENSSSCG00000038055 | 100621483 | 3 | CORO1A | protein_coding |
|  | ENSSSCG00000033744 |  | 3 |  | protein_coding |
|  | ENSSSCG00000035561 | 100621669 | 3 |  | protein_coding |
|  | ENSSSCG00000021557 | 396640 | 3 | SULT1A3 | protein_coding |
|  | ENSSSCG00000022778 | 100627492 | 3 | SGF29 | protein_coding |
|  | ENSSSCG00000040162 | 110260086 | 3 | NUPR1 | protein_coding |
|  | ENSSSCG00000039300 | 493187 | 3 | IL27 | protein_coding |
|  | ENSSSCG00000033623 | 102163208 | 3 |  | protein_coding |
|  | ENSSSCG00000036603 | 100127354 | 3 | CLN3 | protein_coding |
|  | ENSSSCG00000034927 | 110260088 | 3 |  | protein_coding |
|  | ENSSSCG00000021845 | 100515946 | 3 | ATXN2L | protein_coding |
|  | ENSSSCG00000007803 | 100516488 | 3 | TUFM | protein_coding |
|  | ENSSSCG00000007804 | 100516788 | 3 | SH2B1 | protein_coding |
|  | ENSSSCG00000007805 | 100516962 | 3 | ATP2A1 | protein_coding |
|  | ENSSSCG00000007806 | 100517273 | 3 | RABEP2 | protein_coding |
| **HSUA** | ENSSSCG00000031666 | 100511737 | 2 | ZFTA | protein_coding |
|  | ENSSSCG00000013052 | 100512087 | 2 |  | protein_coding |
|  | ENSSSCG00000013054 | 100512265 | 2 | ATL3 | protein_coding |
|  | ENSSSCG00000026914 | 100512584 | 2 | PLAAT3 | protein_coding |
|  | ENSSSCG00000013056 | 100217395 | 2 | LGALS12 | protein_coding |
|  | ENSSSCG00000061988 | 100627034 | 2 | PLAAT5 | protein_coding |
|  | ENSSSCG00000013057 | 110255291 | 2 |  | protein_coding |
|  | ENSSSCG00000027453 | 102164585 | 2 |  | protein_coding |
|  | ENSSSCG00000028537 | 100513779 | 2 |  | protein_coding |
|  | ENSSSCG00000029248 | 100737764 | 2 |  | protein_coding |
|  | ENSSSCG00000060619 |  | 2 |  | protein_coding |
|  | ENSSSCG00000061902 | 100514700 | 2 |  | protein_coding |
|  | ENSSSCG00000025413 | 100737875 | 2 |  | protein_coding |
|  | ENSSSCG00000049137 |  | 2 |  | lncRNA |
|  | ENSSSCG00000038346 |  | 2 |  | lncRNA |
|  | ENSSSCG00000029516 | 407774 | 2 | SLC22A8 | protein_coding |
|  | ENSSSCG00000023571 | 397223 | 2 | SLC22A6 | protein_coding |
|  | ENSSSCG00000040074 |  | 2 |  | protein_coding |
|  | ENSSSCG00000045138 |  | 2 |  | lncRNA |
|  | ENSSSCG00000024545 |  | 2 |  | pseudogene |
|  | ENSSSCG00000022404 | 100144589 | 2 | SLC3A2 | protein_coding |
|  | ENSSSCG00000049213 |  | 2 | U2 | snRNA |
|  | ENSSSCG00000043175 |  | 2 |  | lncRNA |
|  | ENSSSCG00000026837 |  | 2 | SNORD26 | snoRNA |
|  | ENSSSCG00000028290 |  | 2 | SNORD27 | snoRNA |
|  | ENSSSCG00000029095 |  | 2 | SNORD28 | snoRNA |
|  | ENSSSCG00000029549 |  | 2 | SNORD22 | snoRNA |
|  | ENSSSCG00000026212 |  | 2 | SNORD29 | snoRNA |
|  | ENSSSCG00000023669 |  | 2 | SNORD30 | snoRNA |
|  | ENSSSCG00000027032 |  | 2 | SNORD31 | snoRNA |
|  | ENSSSCG00000025513 |  | 2 | SNORD22 | snoRNA |
|  | ENSSSCG00000027405 |  | 2 | U2 | snRNA |
|  | ENSSSCG00000028025 | 100627951 | 2 | WDR74 | protein_coding |
|  | ENSSSCG00000045572 |  | 2 | TEX54 | protein_coding |
|  | ENSSSCG00000026293 | 100628048 | 2 | STX5 | protein_coding |
|  | ENSSSCG00000022866 | 100628213 | 2 | NXF1 | protein_coding |
|  | ENSSSCG00000038711 | 100620450 | 2 | TMEM223 | protein_coding |
|  | ENSSSCG00000032696 | 106509327 | 2 | TMEM179B | protein_coding |
|  | ENSSSCG00000020665 | 100620136 | 2 | TAF6L | protein_coding |
|  | ENSSSCG00000025571 | 100620235 | 2 | POLR2G | protein_coding |
|  | ENSSSCG00000037745 |  | 2 |  | protein_coding |
|  | ENSSSCG00000035472 | 100621273 | 2 | TTC9C | protein_coding |
|  | ENSSSCG00000028542 | 100620658 | 2 | HNRNPUL2 | protein_coding |
|  | ENSSSCG00000020823 | 100144883 | 2 | BSCL2 | protein_coding |
|  | ENSSSCG00000032197 | 100620948 | 2 |  | protein_coding |
|  | ENSSSCG00000024917 |  | 2 |  | pseudogene |
|  | ENSSSCG00000023110 | 100621165 | 2 | UBXN1 | protein_coding |
|  | ENSSSCG00000038370 | 100621476 | 2 | UQCC3 | protein_coding |
|  | ENSSSCG00000027847 | 100620846 | 2 | CSKMT | protein_coding |
|  | ENSSSCG00000025282 |  | 2 | SNORA57 | snoRNA |
|  | ENSSSCG00000058339 | 100621377 | 2 | C11orf98 | protein_coding |
|  | ENSSSCG00000028275 | 100621046 | 2 | INTS5 | protein_coding |
|  | ENSSSCG00000030653 | 396938 | 2 | GANAB | protein_coding |
|  | ENSSSCG00000020809 | 397642 | 2 | B3GAT3 | protein_coding |
|  | ENSSSCG00000033054 | 100622038 | 2 | ROM1 | protein_coding |
|  | ENSSSCG00000013061 | 100515768 | 2 | EML3 | protein_coding |
|  | ENSSSCG00000013062 | 100515941 | 2 | MTA2 | protein_coding |
|  | ENSSSCG00000013063 | 100516127 | 2 | TUT1 | protein_coding |
|  | ENSSSCG00000013064 | 397022 | 2 |  | protein_coding |
|  | ENSSSCG00000036669 |  | 2 |  | protein_coding |
|  | ENSSSCG00000033010 |  | 2 |  | protein_coding |
|  | ENSSSCG00000055375 |  | 2 |  | protein_coding |
| **HSUB** | ENSSSCG00000002752 | 100519194 | 6 | ZFHX3 | protein_coding |
|  | ENSSSCG00000058146 |  | 6 |  | lncRNA |
|  | ENSSSCG00000057766 |  | 6 |  | lncRNA |
|  | ENSSSCG00000060102 |  | 6 |  | lncRNA |
|  | ENSSSCG00000057093 |  | 6 |  | lncRNA |
|  | ENSSSCG00000059221 |  | 6 |  | lncRNA |
|  | ENSSSCG00000053114 |  | 6 |  | lncRNA |
|  | ENSSSCG00000057921 |  | 6 |  | lncRNA |
|  | ENSSSCG00000048469 |  | 6 |  | protein_coding |
|  | ENSSSCG00000047359 |  | 6 |  | lncRNA |
|  | ENSSSCG00000041519 |  | 6 |  | lncRNA |
|  | ENSSSCG00000041474 |  | 6 |  | lncRNA |
|  | ENSSSCG00000056956 |  | 6 |  | lncRNA |
|  | ENSSSCG00000061838 |  | 6 | U6 | snRNA |
|  | ENSSSCG00000063114 |  | 6 |  | lncRNA |
|  | ENSSSCG00000059764 |  | 6 |  | lncRNA |
|  | ENSSSCG00000053257 |  | 6 |  | lncRNA |
|  | ENSSSCG00000061174 |  | 6 |  | lncRNA |
|  | ENSSSCG00000055852 |  | 6 |  | lncRNA |
|  | ENSSSCG00000052407 |  | 6 |  | lncRNA |
|  | ENSSSCG00000048283 |  | 6 |  | lncRNA |
|  | ENSSSCG00000053189 |  | 6 |  | lncRNA |
|  | ENSSSCG00000060193 |  | 6 |  | lncRNA |
|  | ENSSSCG00000054484 |  | 6 |  | lncRNA |
|  | ENSSSCG00000058326 |  | 6 |  | lncRNA |
|  | ENSSSCG00000058647 |  | 9 |  | lncRNA |
|  | ENSSSCG00000034240 |  | 9 |  | protein_coding |
|  | ENSSSCG00000053914 |  | 9 |  | lncRNA |
|  | ENSSSCG00000058845 |  | 9 |  | lncRNA |
|  | ENSSSCG00000047651 |  | 9 |  | lncRNA |
|  | ENSSSCG00000063052 |  | 9 |  | lncRNA |
|  | ENSSSCG00000043081 |  | 9 |  | lncRNA |
|  | ENSSSCG00000060212 |  | 9 |  | lncRNA |
|  | ENSSSCG00000054810 |  | 9 |  | lncRNA |
|  | ENSSSCG00000058369 |  | 9 |  | lncRNA |
|  | ENSSSCG00000061625 |  | 9 |  | lncRNA |
|  | ENSSSCG00000061802 |  | 9 |  | lncRNA |
|  | ENSSSCG00000057296 |  | 9 |  | lncRNA |
|  | ENSSSCG00000055654 |  | 9 |  | lncRNA |
|  | ENSSSCG00000063053 |  | 9 |  | lncRNA |
|  | ENSSSCG00000056331 |  | 9 |  | lncRNA |
|  | ENSSSCG00000052974 |  | 9 |  | lncRNA |
|  | ENSSSCG00000055230 |  | 9 |  | lncRNA |
|  | ENSSSCG00000059874 |  | 9 |  | lncRNA |
|  | ENSSSCG00000057734 |  | 9 |  | lncRNA |
|  | ENSSSCG00000054322 |  | 9 |  | lncRNA |
|  | ENSSSCG00000063012 |  | 9 |  | lncRNA |
|  | ENSSSCG00000059818 |  | 9 |  | lncRNA |
|  | ENSSSCG00000056809 |  | 9 |  | lncRNA |
|  | ENSSSCG00000063131 |  | 9 |  | lncRNA |
|  | ENSSSCG00000056937 |  | 9 |  | lncRNA |
|  | ENSSSCG00000045408 |  | 9 |  | lncRNA |
|  | ENSSSCG00000045017 |  | 9 |  | lncRNA |
|  | ENSSSCG00000050782 |  | 9 |  | lncRNA |
|  | ENSSSCG00000048934 |  | 9 |  | lncRNA |
|  | ENSSSCG00000058844 |  | 9 |  | lncRNA |
|  | ENSSSCG00000015310 | 396908 | 9 | AKAP9 | protein_coding |
|  | ENSSSCG00000015311 | 403334 | 9 | CYP51A1 | protein_coding |
|  | ENSSSCG00000036187 |  | 9 | U6 | snRNA |
|  | ENSSSCG00000029092 | 102167908 | 9 | LRRD1 | protein_coding |
|  | ENSSSCG00000031827 |  | 9 |  | protein_coding |
|  | ENSSSCG00000015313 | 100517669 | 9 | KRIT1 | protein_coding |
|  | ENSSSCG00000015314 | 100517844 | 9 | ANKIB1 | protein_coding |
|  | ENSSSCG00000059298 |  | 9 | U6 | snRNA |
|  | ENSSSCG00000027356 |  | 9 |  | protein_coding |
|  | ENSSSCG00000031835 | 100627096 | 9 | GATAD1 | protein_coding |
|  | ENSSSCG00000029967 | 100626142 | 9 | PEX1 | protein_coding |
|  | ENSSSCG00000015315 | 100518743 | 9 | RBM48 | protein_coding |
|  | ENSSSCG00000015316 | 100519264 | 9 |  | protein_coding |
|  | ENSSSCG00000045005 | 100519438 | 9 | FAM133B | protein_coding |
|  | ENSSSCG00000040183 |  | 9 | CDK6 | protein_coding |
|  | ENSSSCG00000058359 |  | 9 |  | lncRNA |
|  | ENSSSCG00000027372 | 100519098 | 9 | SAMD9 | protein_coding |
|  | ENSSSCG00000025870 | 100626473 | 9 | HEPACAM2 | protein_coding |
| **HSD** | ENSSSCG00000058647 |  | 9 |  | lncRNA |
|  | ENSSSCG00000034240 |  | 9 |  | protein_coding |
|  | ENSSSCG00000053914 |  | 9 |  | lncRNA |
|  | ENSSSCG00000058845 |  | 9 |  | lncRNA |
|  | ENSSSCG00000047651 |  | 9 |  | lncRNA |
|  | ENSSSCG00000063052 |  | 9 |  | lncRNA |
|  | ENSSSCG00000043081 |  | 9 |  | lncRNA |
|  | ENSSSCG00000060212 |  | 9 |  | lncRNA |
|  | ENSSSCG00000054810 |  | 9 |  | lncRNA |
|  | ENSSSCG00000058369 |  | 9 |  | lncRNA |
|  | ENSSSCG00000061625 |  | 9 |  | lncRNA |
|  | ENSSSCG00000061802 |  | 9 |  | lncRNA |
|  | ENSSSCG00000057296 |  | 9 |  | lncRNA |
|  | ENSSSCG00000055654 |  | 9 |  | lncRNA |
|  | ENSSSCG00000063053 |  | 9 |  | lncRNA |
|  | ENSSSCG00000056331 |  | 9 |  | lncRNA |
|  | ENSSSCG00000052974 |  | 9 |  | lncRNA |
|  | ENSSSCG00000055230 |  | 9 |  | lncRNA |
|  | ENSSSCG00000059874 |  | 9 |  | lncRNA |
|  | ENSSSCG00000057734 |  | 9 |  | lncRNA |
|  | ENSSSCG00000054322 |  | 9 |  | lncRNA |
|  | ENSSSCG00000063012 |  | 9 |  | lncRNA |
|  | ENSSSCG00000059818 |  | 9 |  | lncRNA |
|  | ENSSSCG00000056809 |  | 9 |  | lncRNA |
|  | ENSSSCG00000063131 |  | 9 |  | lncRNA |
|  | ENSSSCG00000056937 |  | 9 |  | lncRNA |
|  | ENSSSCG00000045408 |  | 9 |  | lncRNA |
|  | ENSSSCG00000045017 |  | 9 |  | lncRNA |
|  | ENSSSCG00000050782 |  | 9 |  | lncRNA |
|  | ENSSSCG00000048934 |  | 9 |  | lncRNA |
|  | ENSSSCG00000058844 |  | 9 |  | lncRNA |

**^1^CHR**: Chromosome number
